# Optimization of Mopan Persimmon Wine Fermentation with Pectinase and Analysis of Its Mechanism of Action

**DOI:** 10.3390/foods12061246

**Published:** 2023-03-15

**Authors:** Zijuan Wang, Qinghong Hao, Xiaowen An, Bimal Chitrakar, Jiamin Li, Zhihui Zhao, Changwei Ao, Jinxu Sun

**Affiliations:** 1 College of Food Science and Technology, Hebei Agricultural University, Baoding 071000, China; 2 College of Life Science, Hebei Agricultural University, Baoding 071000, China; 3 College of Horticulture, Hebei Agricultural University, Baoding 071000, China; 4 Hebei Technology Innovation Center for Fruits and Vegetables Fermentation, Hengshui 053000, China

**Keywords:** Mopan persimmon, persimmon wine, pectinase, physicochemical properties, SEM

## Abstract

Due to the high sugar content of Mopan persimmon, which has an annual output of more than 0.5 million tons in China, it can be processed to make fruit wine. In this study, a strain of yeast screened from different persimmon samples was used for persimmon wine fermentation. The optimal conditions of persimmon wine fermentation were determined through single-factor experiments as follows: Yeast addition of 0.08 g/kg; a fermentation temperature of 28 °C; sucrose addition of 18%; and pectinase addition of 0.01%. Under these conditions, the alcohol content of persimmon wine reached 12.9%. The addition of pectinase during persimmon wine fermentation was found to decompose pectin at high speed, reduce the viscosity of the fermentation liquid, increase the dissolved oxygen content in the fermentation liquid, promote the growth and reproduction of yeast, and effectively convert the sugars into alcohol. After fermentation, alcohol, residual sugars, and total phenolic content with or without pectinase treatment were 12.9 and 4.4%, 2.2 and 13.4 g/L, and 738.7 and 302 µg/mL, respectively. Scanning electron microscopy (SEM) results showed that compared with the mash without pectinase treatment, the mash with pectinase had a larger network structure and more pores and yeasts.

## 1. Introduction

Persimmon (*Diospyros* spp.) belongs to the Ebenaceae family, which contains approximately 500 species of trees and shrubs, of which more than 350 species are distributed in tropical and subtropical regions worldwide [1,2]. The global annual production of persimmon exceeded 5.75 million tons [3], with a rising trend in demand and consumption. Persimmon is beneficial to health and contains high amounts of phenolic compounds, carotenoids, fiber, vitamins, and minerals with a high antioxidant capacity [4,5,6]. Xiaowen An et al. [7] reported that fermented persimmon beverages contain a lot amount of organo-oxygen compounds, prenol lipids, fatty acyls, flavonoids, carboxylic acids, and derivatives.

The Mopan persimmon used in this experiment is mainly produced in the northern part of the Tai-hang Mountain region and the southern part of the Yan-shan Mountain region in northern China with an annual output of more than 0.5 million tons [8]. The average Mopan persimmon fruit weighs more than 250 g and its soluble solid content is about 15%, which is conducive to the production of alcohol. There are different ways of persimmon processing to produce value-added end products, such as jams [9], wines [10], juices [11], dried products [12], and vinegar [13].

There are many things that affect the fermentation of fruit wines, including the amount of yeast addition, the fermentation time and temperature, etc. [14]. Tian et al. [15] showed that the optimal conditions for fermented 354 greengage wine were determined to be a fermentation temperature of 20 °C, a material–liquid 355 ratio of 1:2.0, and a sugar content of 180 g/L. Under this optimal condition, the alcohol content of the wine reached 12.5%. Panesar et al. [16] reported the fermentation conditions as 26 °Brix total soluble solids, 5.4 pH, 29 °C temperature, 7.5% inoculum size, and 5 days of fermentation. The fermentation temperature has a great influence on the fermentation because yeast growth and reproduction are related to the fermentation temperature, which directly affects the fermentation efficiency. When the fermentation temperature is low, yeast growth is slow and the rate of sugar fermentation to produce alcohol is slow, making the fermentation time longer and leading to inadequate fermentation. Conversely, when the fermentation temperature is high, the yeast grows faster and the fermentation completes earlier, making the amount of alcohol produced by sugar conversion lower [17]. The addition of pectinase was found to induce remarkable increases in the predominant phenolic compounds and antioxidant activity in persimmon wine [18]. Jiang et al. [19] showed that pectinase treatment increased the total phenolic content and enriched esters and terpenes in red dragon fruit wine.

Despite the high sugar content of persimmons, the production of persimmon wine is more challenging than that of grape wine. Currently, the main problem for persimmon wine companies in Baoding is that, during fermentation, the yeast quickly dies, which leads to a halt in the utilization of persimmon sugar, resulting in a higher residual sugar content (over 15%) and a lower alcohol content (around 5%). This ultimately results in an easy growth of acetic acid bacteria with the formation of a higher content of volatile acids. The physical state of the persimmon after crushing is much different from that of grapes, apples, and other fruits. The latter fruits are in a liquid state after crushing, while, because persimmon contains a high amount of pectin, its crushed pulp is highly viscous. Pectin makes the fermentation process harder because of its fiber-like molecular structure. Pretreatment is needed for persimmon mash to degrade pectin and soften the crushed material before wine fermentation [18]. Pectinase decomposes the polysaccharide matrix on the plant cell walls, increases the self-flow of juice; decomposes pectin into small molecular compounds, softens the pulp tissue; reduces the pulp viscosity, improves the crushing performance, and increases the juice yield [20]. In the brewing progress of wine, the use of pectinase solves problems of turbidity and sediment formation, improves the transmittance of wine, maintains the stability of wine quality, increases the leaching of natural pigments in the wine, and improves the phenolic content [21]. The role of pectinase during winemaking is mostly to improve the yield in wine and to improve wine clarification [22]. However, few people have analyzed the effect of adding pectinase on yeast growth during fermentation [23].

At present, due to the lower demand for fresh persimmon fruit in the northern mountainous areas of China, approximately half of the fresh fruits are spoiled in the orchards every year. The utilization rate of persimmon can be improved by processing it into wine that can be stored for a few years. Persimmon wine has a long history of production; however, due to the stickiness of the fruit, the fermentation efficiency is low and the quality of persimmon wine is much poorer than that of grape wine. The aim of this study was to optimize the fermentation conditions of persimmon wine, which includes yeast screening, fermentation temperature, sucrose addition, and yeast addition. Furthermore, ways to improve fermentation efficiency with or without pectinase were explored and the physicochemical parameters of the broth during fermentation were compared. We expect this research to provide a foundation for the production of high-quality persimmon wine.

## 2. Materials and Methods

### 2.1. Materials and Reagents

Mature Mopan persimmons (soluble solids ≥15 °Brix; single fruit weight ≥ 200 g; and total sugar ≥9 g/100 g) were collected from a persimmon orchard in Kangguan Village, Shunping County, Hebei Province and stored at 4 °C until the fruit was processed (within two months). Pectin lyase was obtained from Novozyme (Novozyme, Demark), consisting mainly of polygalacturonase, pectin trans-eliminase, and pectin esterase. Forinol and galacturonic acid were obtained from Sigma-Aldrich (Shanghai, China). Potassium sodium tartrate, potassium ferricyanide, phenolphthalein, and glucose were obtained from Fuchen Chemical Reagent Co. (Tianjin, China).

### 2.2. Yeast Screening

#### 2.2.1. Yeast Separation and Purification

More than 200 persimmon samples were selected from orchards within a 100 square kilometer range in the Taihang Mountains region of Baoding, Hebei, China. A portion of the fruit flesh was placed in YPD liquid medium at 28 °C for 24 h to allow the yeast to fully grow in the liquid culture. A small amount of the culture was then diluted with sterile water and spread on YPD solid medium, which was incubated in an incubator at 28 °C for 48 h. Smooth, moist, single colonies with typical yeast strain morphology were selected and further cultured until pure colonies were screened out.

#### 2.2.2. Determination of Fermentation Performance of Yeast

A series of fermentation performance tests were conducted on the selected yeast, which was compared to commercially available Angel yeast (BV818, Yichang, China). The selected yeast was inoculated into YPD culture medium with a soluble solids content of approximately 15 °Brix at a 5% (*v*/*v*) addition rate. The soluble solids content was determined daily at 25 °C until the end of fermentation, at which point, the ethanol content was also determined.

### 2.3. Persimmon Winemaking

Briefly, manually sorted Mopan persimmon fruit were washed and drained. Then, the fruit was softened with CO_2_ (20 °C for 7 days) and crushed to pulp, to which sucrose (15%, *w*/*v*) was added to adjust the potential final alcoholic degree to 10–15% by volume during vigorous fermentation. The slurry was then treated with pectinase (0.01%, *w*/*v*; Novozyme, Demark). To improve the efficiency of the pectinase, the slurry was stirred for 30 min during treatment. To prepare the yeast-activated liquid, a freeze-dried yeast powder (0.08 g/kg, *w*/*w*) was weighed and added to 10 times the volume of sterile 5% sugar water at 35 °C, incubating for 15 min. The yeast count was approximately 48.75 × 10^6^ CFU/mL. Activated yeast and 0.2 g/L potassium metabisulfite were inoculated for fermentation at 28 °C for approximately 5–7 days. When the content of total sugar of the persimmon mash dropped below 4.0 g/L, fermentation was considered to be completed. After alcoholic fermentation, persimmon wine was filtered by using a 120 mesh filter cloth and stored at 4 °C for further analysis.

### 2.4. Optimization of Mopan Persimmon Wine Fermentation

According to previous methods [24,25,26] and existing research in the laboratory, a preliminary investigation of the factors affecting the taste and quality of fermented Mopan persimmon wine was conducted using fermentation temperature, sucrose concentration, pectinase, and yeast addition. The factors chosen were the sucrose addition (6, 10, 14, 18, and 22%), yeast addition (0.02, 0.04, 0.06, 0.08, and 0.1%), pectinase addition (0, 0.005, 0.010, 0.015, and 0.020%), fermentation temperature (20, 24, 28, 32, and 36 °C). Five kilograms of persimmon fruit was taken for fermentation at each treatment, and each measurement was repeated three times. At the end of fermentation, the content of soluble solids, alcohol, reducing sugar, total sugar, and acid of Mopan persimmon wine was measured to determine the appropriate fermentation process.

### 2.5. Determination of Physicochemical Parameters

Soluble solids were determined with a refractometer (PAL-1, Aitco, Japan). Viscosity was measured using a viscometer (NDJ-5S, Shanghai, China). The dissolved oxygen content was measured with a magnetic stirring electrode (JPB-607A, Shanghai, China). According to the instructions for the oxygen electrode, the value of the oxygen electrode was 100% in air and 0% in saturated potassium sulfate solution. The number of yeast cells was measured using a blood-counting chamber [27]. Reducing sugar and total sugar values were determined by the Fehling titration method [28]. Titratable acid was determined by acid-base titration, which was calculated as tartaric acid [29].

### 2.6. Analysis of Ethanol with GC-FID

Analysis of alcohol content was performed by using a gas chromatograph (GC, 7890A, Agilent Technologies, CA, USA) equipped with a hydrogen flame ionization detector (FID) according to the procedure described by Cassiano et al. [30] with slight modifications. The column used was a TG-5MS capillary column (30 m × 0.25 mm × 0.25 μm). The oven temperature was held at 60 °C for 3 min, then increased to 100 °C at a rate of 3 °C/min and held at 100 °C for 2 min. The column temperature was 200 °C and the temperature in the gasification chamber and detector was 240 °C. The injection volume was 1.0 μL, the flow rate was 40 mL/min, the airflow rate was 400 mL/min, and the carrier gas (N_2_) flow rate was 40 mL/min. The linear regression equation of the ethanol standard curve was Y = 104,390X − 8.1504, the correlation coefficient was R^2^ = 0.9987, and the calibration range of ethanol was 1.00–12.50%vol.

### 2.7. Total Phenolic Content Measurement

The total phenolic content was measured at 765 nm through a UV-Visible spectrophotometer (TU-1810, Beijing, China) according to the Folin–Ciocalteu method as reported by Singleton and Rossi [31]. Results were expressed as gallic acid equivalents (mg/L). A calibration line was built on the basis of solutions at known and increasing concentrations of gallic acid. Three measurements were performed for each sample. The standard curve of total phenolics was Y = 0.0184X + 0.0318, the correlation coefficient was R^2^ = 0.9978, and the calibration range of total gallic acid was 0–50 mg/L. The total phenolic content was calculated using the standard curve of gallic acid.

### 2.8. Pectin Content Analysis

The total pectin content of persimmon wine was determined spectrophotometrically by the method of McCready and McComp [32]. Wine (15 mL) was put into a 50 mL centrifuge tube, 25 mL of 95% ethanol solution was added, the mixture was heated in a water bath at 85 °C for 10 min with thorough stirring and then, 95% ethanol was added to make the solution approximate 50 mL. After centrifugation at 3000 r/min for 15 min, the precipitate was collected, which was dissolved in a 100 mL volumetric flask with 63% ethanol. Then, 5 mL of 1 mol/L sodium hydroxide solution was added and volume was made to the scale with distilled water and mixed well. Pectin substances were extracted and then reacted with carbazole in acidic media. The absorbance of the colored solution was determined at 525 nm. The pectin content of the samples was expressed as the amount of galacturonic acid. The linear regression equation of the calibration curve of galacturonic acid was Y = 0.0073X − 0.1112, the correlation coefficient was R^2^ = 0.9603, and the calibration range of galacturonic acid was 20–100 g/L.

### 2.9. SEM Analysis of the Pulp during Fermentation

To study the effect of pectinase on the fermented mash, the surface structure of vacuum freeze-dried Mopan persimmon mash was observed by VEGA 3 SBH/EDX (USA) scanning electron microscopy [33]. Samples used for scanning electron microscopy were taken from the first, second, and third days of fermentation. The vacuum-freeze-dried samples were placed on the carbon conductive belt of the sample shelf and the dried mash was pressed to ensure that no gaps were left between the particles. The loose particles were removed by an atomizer and coated with gold film by an ion sputter device (GSL-1100X-SPC-12, China). Such samples were placed under the scanning electron microscope for observation. Images were obtained at 300× and 1000× magnification.

### 2.10. Statistical Analysis

All experiments were performed with three replications. Statistical analysis was carried out by one-way ANOVA (Analysis of variance) and Tukey’s test at a 95% confidence level using SPSS 25.0 software for Windows (SPSS Inc., Chicago, IL, USA). The results were considered statistically significant when *p* < 0.05.

## 3. Results and Discussion

### 3.1. Yeast Strain Screening

A total of 427 single colonies of yeast were isolated and purified from samples collected from persimmons from different regions around Baoding. Among them, 10 strains with strong activity were selected for further testing. As shown in Table 1, there were significant differences in the soluble solids and ethanol content of the wine fermented with these 10 yeast strains; all showed strong fermentation capabilities. Since strain F9 showed the best performance with the lowest residual sugar content and the highest alcohol content after 6 days of fermentation, it was selected as the fermentation strain for persimmon wine. Based on previous research and laboratory results, the selected strain F9 was cultured on a larger scale, collected by centrifugation, and treated with a protective agent, namely trehalose (Hayashibara Co., Ltd., Okayama, Japan) for freeze-drying. The freeze-dried sample was stored at 4 °C for use in further persimmon wine fermentation. The freeze-dried yeast should be appropriately crushed before use.

### 3.2. Optimal Fermentation Conditions for Mopan Persimmon Wine

#### 3.2.1. Yeast Addition

**Figure 1:**
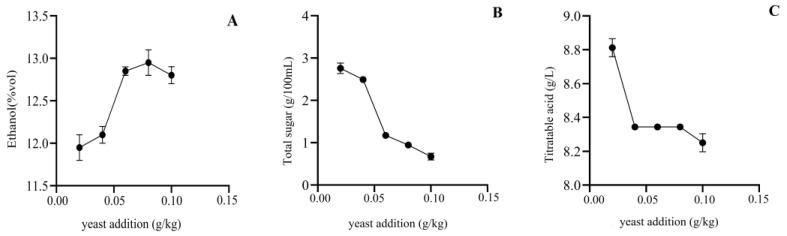
The effects of yeast addition on alcohol (**A**), total sugar (**B**), and titratable acid (**C**) content of Mopan persimmon wine.

With the fermentation temperature fixed at 28 °C, sucrose addition at 18%, and pectinase addition at 0.010%, the alcohol, total sugar, and total acid content of persimmon wine produced using the above fermentation process were measured for different amounts of yeast (Figure 1). As the amount of yeast increased, the alcohol content of the persimmon wine initially rose and then decreased, reaching a maximum of 12.95% at the yeast addition of 0.08 g/kg. The total sugar content showed a trend of decreasing initially and then increasing with the amount of yeast addition, showing the lowest total sugar content when the alcohol content was the highest. The total acid content generally decreased. Fermentation of wine involves the conversion of sugar to alcohol and CO_2_ by yeast. The amount of yeast added can affect the quality of the wine. When the yeast addition is appropriate, all of the sugar can be converted to alcohol. When the yeast addition is too low, the growth and reproduction of the yeast is slow and its ability to convert sugar to alcohol is weak, resulting in an incomplete fermentation, high residual sugar, and low alcohol content in the wine with an increased risk of the growth of contaminants and acid-producing bacteria that can degrade the quality of the wine. Conversely, when the yeast addition is too high, the growth of the yeast consumes a large amount of sugar, reducing the amount of sugar available for the production of ethanol, resulting in less ethanol produced and excess yeast remaining in the wine, which can affect the aroma and clarity of the wine [34]. Therefore, a yeast addition of 0.08 g/kg was considered suitable in this study.

#### 3.2.2. Sucrose Addition

**Figure 2:**
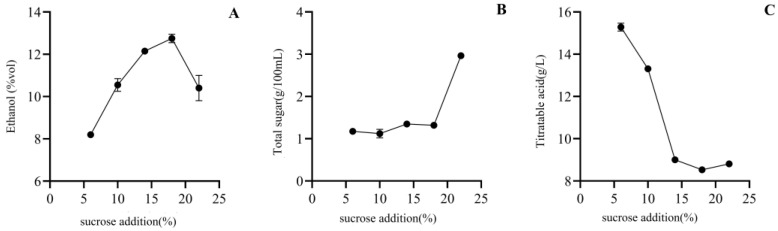
The effects of sucrose addition on alcohol (**A**), total sugar (**B**), and titratable acid (**C**) content of Mopan persimmon wine.

By fixing the yeast addition at 0.08 g/kg, the fermentation temperature at 28 ℃, and the pectinase addition at 0.010%, the results of alcohol, total sugar, and total acid content in the persimmon wine produced by changing the added sugar content are shown in Figure 2. As the sugar addition increased, the alcohol content of the wine initially increased and then decreased, with the highest alcohol content of 12.75% at a sugar level of 18%. The total sugar content generally increases with increasing sugar addition, while the total acidity generally decreases. The sugar addition can directly affect the alcohol content of the wine. Some fruits have a low sugar content, which is insufficient to be converted into the required alcohol content; so, additional sugar is needed to increase the alcohol content. When the sugar addition is low, the yeast has a lower amount of nutrients available for growth and reproduction, leading to slow metabolism and low fermentation efficiency as well as low sugar content and high acidity. Conversely, when the sugar addition is too high, yeast growth is inhibited, which is not conducive to later fermentation [35]. Therefore, a sugar addition of 18% was considered appropriate.

#### 3.2.3. Pectinase Addition

**Figure 3:**
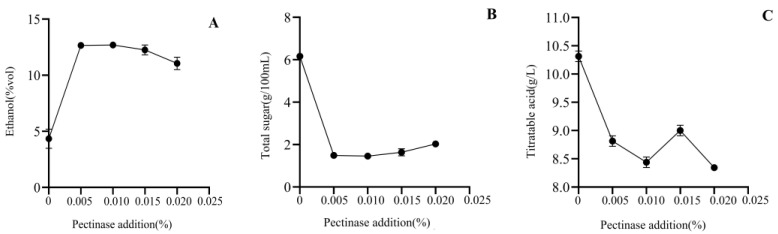
The effects of pectinase addition on alcohol (**A**), total sugar (**B**), and titratable acid (**C**) content of Mopan persimmon wine.

With a fixed yeast addition of 0.08 g/kg, sucrose addition of 18%, and a fermentation temperature of 28 °C, the results of alcohol, total sugar, and total acid content of the Mopan persimmon wine obtained by changing the amount of pectinase are shown in Figure 3. As the amount of pectinase increased, the alcohol content of the persimmon wine first increased and then slowly decreased. When the pectinase addition was 0.005%, the alcohol content was 12.65%; when the pectinase addition was increased to 0.01%, the highest alcohol content was observed (12.70%). The total sugar content of the persimmon wine decreased first and then slowly increased with the increase in pectinase addition, while the total acid content showed a general downward trend. The wine fermented without pectinase had the lowest alcohol content and the highest acid content. Compared with other fruits, the Mopan persimmon juice was more viscous, had lower fermentation efficiency, and was prone to grow bacteria that produce acid, which might reduce the quality of the wine. Adding pectinase reduced the viscosity of the juice and improved the fermentation efficiency. Therefore, a pectinase addition of 0.01% was selected.

#### 3.2.4. Fermentation Temperature Optimization

With a fixed amount of yeast added of 0.08 g/kg, of sucrose added of 18%, and of pectinase added of 0.01%, the results of the alcohol, total sugar, and total acid content of the Mopan persimmon wine obtained by changing the fermentation temperature are shown in Table 2.

As shown in Table 2, the alcohol content in the wine was between 8.20 and 12.75%, titratable acidity ranged between 7.97 and 12.19 g/L, and residual sugar ranged between 0.53 and 1.85 g/100 mL. Titratable acidity constantly increased, while, residual sugar slightly decreased, with increasing temperature in the experimental range of 20 to 28 °C. Therefore, the fermentation temperature had different effects on all five physicochemical parameters. Acidity plays an important biotechnological role in determining the stability and sensory characteristics of persimmon wine [36]. Due to insufficient fermentation, low alcohol content and high fermentation temperature, as well as slow bacterial growth and high wine acidity, results [37]. Titratable acidity is the total amount of acid in persimmon wine. Some acids in persimmon wine are naturally present in persimmon, while others are byproducts of fermentation (volatile acidity: 0.8–1.6 g/L). Acidity greatly influences the taste of persimmon wine and the flavor develops during aging. High titratable acidity also has detrimental effects on the sensory quality of the wine. Alcohol is a key element of wine quality, which is fundamental for organoleptic properties and stability and acts as a solvent for aromas [38]. The alcohol content in the tested samples slightly increased with the fermentation temperature from 20 to 28 °C and gradually decreased between 28 and 36 °C. The alcohol strength showed the highest level of 12.75% at the fermentation temperature of 28 °C. At low temperatures of 20, 24, and 28 °C, the alcohol content was slightly higher, which might be caused by the higher cell death induced by higher temperatures. This result also agreed with the findings reported by Torija et al. [39]. Since the alcohol in wine is converted from sugar, the sugar content was lower when the alcohol content of wine was higher. When the fermentation temperature was too high, the yeast proliferation was accelerated and the bacteria entered the decaying period too early, which was not conducive to the conversion of sugar into alcohol, resulting in insufficient post-fermentation. Therefore, the optimum fermentation temperature of Mopan persimmon wine was 28 °C.

### 3.3. Mechanism Analysis of the Pectinase Effect on Fermentation

#### 3.3.1. Soluble Solids Changes with Pectinase Treatment

**Figure 4:**
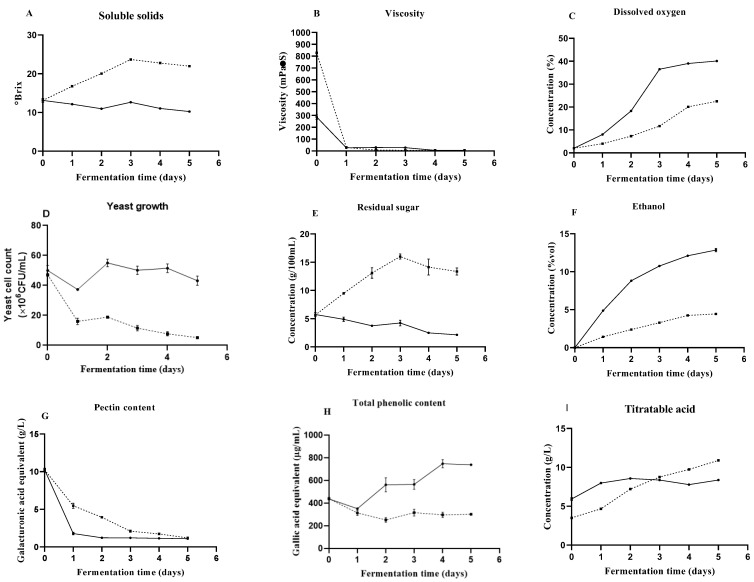
Changes in (**A**) soluble solids, (**B**) viscosity, (**C**) dissolved oxygen, (**D**) yeast growth, (**E**) residual sugar, (**F**) ethanol (**G**) pectin content, (**H**) total phenolic content and (**I**) titratable acid of Mopan persimmon mash during fermentation. Pectinase treatment (—●—) and control without pectinase treatment (--■--). Values are given as the means ± standard deviations (*n* = 3).

The change in soluble solids in Mopan persimmon mash was determined by a refractometer during fermentation (Figure 4A). Since 15% (*w*/*v*) sucrose was added to the Mopan persimmon mash during the first three days, the soluble solids of the mash did not decrease quickly or rise. The results showed that the soluble solids content of persimmon mash with pectinase decreased significantly at the beginning of fermentation and stabilized at the end of fermentation, while the soluble solids content of persimmon mash without pectinase gradually increased and then decreased slowly during fermentation. The content of soluble solids in wine with pectinase was significantly lower than that without pectinase, indicating that the addition of pectinase would have an effect on the fermentation process and the content of soluble solids in wine. The soluble solids of mash with pectinase decreased significantly with prolonged fermentation time (*p* < 0.05). By analyzing the fermentation process, after the beginning of the fermentation of Mopan persimmon pulp, most of the sugar was successfully converted into alcohol, but yeast also had a certain inhibitory effect with the improvement in alcohol content [40]. Overall, the soluble solids content showed a downward trend, as did the total sugar content. Under appropriate fermentation conditions, the total sugar decreased to a certain extent and remained at a stable level when the fermentation was completed [41].

#### 3.3.2. Effect of Pectinase Treatment on Yeast Growth and Oenological Properties

The viscosity of persimmon mash (286.0 mPa•S) with pectinase was significantly lower (*p* < 0.05) compared to that (828.7 mPa•S) without pectinase in the early stage of fermentation. Within one day of the initial reaction, the viscosity of the Mopan persimmon mash decreased rapidly (Figure 4B) and on the last day of fermentation, the viscosity remained unchanged (5.7 mPa•S). However, the viscosity of fermentation broth with and without pectinase was almost the same after one day of fermentation. The reason was that the two broths (after one day of fermentation) used to measure viscosity were filtered liquids. At this stage of fermentation, it was difficult to directly measure the viscosity of the fermentation broth. The viscosity of the polymer solution is related to the concentration of the solution, the relative molecular weight of the polymer, and the shape of the polymer molecule when the concentrations of the pulp and the species in the contents remain constant. Therefore, the rapid decrease in viscosity in the fermentation process of Mopan persimmon mash is directly related to the decrease in the relative molecular weight of the polymer polysaccharide in the pulp. Because pectinase is usually composed of pectin lyase, polygalacturonic acid enzyme, and pectin esterase enzyme complex [42], pectin molecular weight reduces quickly and juice viscosity falls rapidly at the initial stage, by the random cutting of juice-suspended particles in the pectin molecule chains of the enzyme solution.

Dissolved oxygen is the absolute concentration of oxygen dissolved in water, which is generally calibrated under atmospheric pressure. According to the instruction of the dissolved oxygen electrode, air and saturated sodium sulfite were used for the calibration. The dissolved oxygen content was 2.0% because of the high viscosity of persimmon pulp when fermentation started. After fermentation for one day, the viscosity of the Mopan persimmon mash decreased and the dissolved oxygen increased gradually. The dissolved oxygen of the mash with pectinase increased from 2.0 to 40.1%, and that without pectinase increased from 2.0 to 22.5% (Figure 4C). Changes in dissolved oxygen affect the whole redox potential of the fermentation system, thus affecting the growth of yeast cells and the synthesis of products [43].

The yeast count decreased gradually on the first day of fermentation (Figure 4D) because of the low dissolved oxygen content. The number of yeast in the enzyme-treated persimmon mash increased to a maximum (55 × 10^6^ CFU/mL) on approximately the second day of fermentation, which was higher than that without pectinase (18.83 × 10^6^ CFU/mL). Afterward, the number of yeast gradually decreased because the yeast entered a stable phase under the conditions of much higher alcohol content and lower reducing sugar content.

Alcoholic fermentation is a process of converting sugar into alcohol. The sugar content in wine is closely related to the fermentation process. Sucrose (15% *w*/*v*) was added to the persimmon mash on the first, second, and third day of fermentation. Therefore, the sugar content of Mopan persimmon mash supplemented with pectinase did not decrease quickly in the first three days of fermentation (Figure 4E). However, the mash without pectinase might not convert sugar into alcohol effectively because of its higher viscosity, lower dissolved oxygen, and lower amounts of yeast. The residual sugar (2.15 g/100 mL) of Mopan persimmon wine supplemented with pectinase was significantly different from that (13.36 g/100 mL) of wine without pectinase (*p* < 0.05). Combined with the variation curve of the number of yeast in persimmon mash, in the mash with pectinase treatment, the number of yeast caused the reducing sugar to ferment into ethanol effectively. However, the wine without pectinase had fewer yeast, and, thus, the fermentation ability was weak, resulting in the accumulation of sugar in the mash.

In the fermentation process of Mopan persimmon pulp with pectinase, the alcoholic strength first increased and then tended to be stable (Figure 4F). After two days of fermentation, the ethanol content increased significantly (*p* < 0.05) from zero to 8.82%. After 3–5 days of fermentation, the ethanol content continuously increased to 12.86% at the end of fermentation. In the fermentation process of mash without pectinase, the alcoholic strength increased slowly from zero to 4.44% at the end of fermentation. According to the calculation in Figure 4E,F, total sugar and its ethanol conversion yields with and without pectinase treatment were about 90% and 38%, respectively. The main reason for the low alcohol content was that the viscosity of mash without pectinase was significantly higher than that with pectinase, which led to the low dissolved oxygen concentration and yeast growth in persimmon mash.

#### 3.3.3. Effect of Pectinase Treatment on Pectin, Total Phenolic, and Titratable Acid Content

Pectin is a kind of high molecular weight polysaccharide compound that is present in almost all plants as part of the cellular structure [3]. The pectin content of the Mopan persimmon mash decreased gradually during the fermentation process (Figure 4G). The pectin content of mash supplemented with pectinase significantly decreased from 10.24 g/L to 1.78 g/L on the first day of fermentation (*p* < 0.05) and then decreased to 1.10 g/L at the end of fermentation. The pectin content of the mash without pectinase decreased gradually during fermentation from 10.24 g/L to 1.20 g/L at the end of fermentation. After fermentation, the pectin content of the wine with pectinase was not significantly different from that without pectinase. In this process, pectin lyase (PL) can break the long chain of pectin and form short-chain pectin molecules [44].

Phenolic profiles are important quality parameters of wine due to their great contributions to organoleptic quality, antioxidant activity, formation of aroma, and colloidal stability [45]. In the fermentation process of Mopan persimmon mash with pectinase, the total phenolic content showed a trend of a gradual increase (Figure 4H). The total phenolic content of persimmon pulp was 437.88 µg/mL before fermentation, which significantly increased to 738.70 µg/mL on the fifth day of fermentation. In the traditional process, beneficial polyphenols (Such as pigments, tannins, etc.) in the cell were hindered by the cell wall in the process of outward diffusion. In this case, the impregnation of pectinase might accelerate the impregnation dissolution of pigments and tannins and improve the stability of pigments and tannins [21]. Pectinase was used to decompose pectin to transform polymerized polyphenolics into free polyphenolics and improve the extraction rate of polyphenolics [46]. However, in the fermentation process of Mopan persimmon pulp without pectinase, the total phenolic content showed a trend of decreasing gradually at first and then becoming stable. The total phenolic content of wine without pectinase treatment was 302.01 µg/mL when fermentation ended.

In the fermentation process, titratable acid content showed an upward trend (Figure 4I). The titratable acid content in the fermentation pulp with pectinase increased significantly on the first day and then gradually stabilized (5.94 to 8.37 g/L), while the titratable acid content in the fermentation pulp without pectinase increased with the progress of fermentation (3.50 to 10.90 g/L). The Persimmon wine fermentation process produces a certain amount of acetic acid and lactic acid. Although it is not the main factor in increasing the total acid, it accounts for a part of the total acid content. A small amount of acetic acid is produced by yeast metabolism and lactic acid may be produced by fermenting sugar or malic acid.

Similar findings in previous studies showed that the addition of pectinase enzymes in fruit juice resulted in increased wine yield; for example, by 16% in red dragon fruit wine [47]. This outcome is even more evident in persimmon wine. It plays important roles in reducing viscosity and increasing yield through the liquefaction of pulp and removal of peel. Depending on the type of fruit and application conditions, the increase in final yields varies.

### 3.4. SEM Analysis of the Persimmon Fermentation

**Figure 5:**
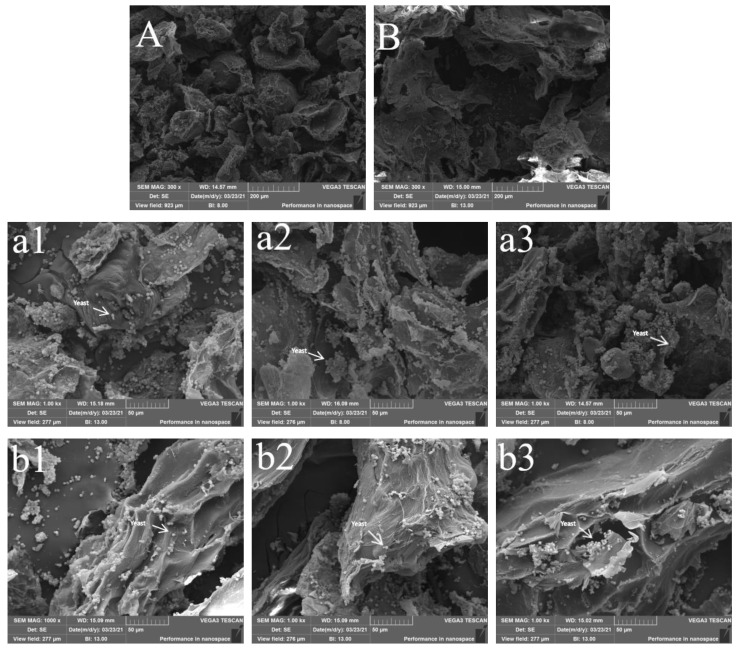
SEM of Mopan persimmon mash with pectinase treatment (**A**,**a1**–**a3**) and persimmon mash without pectinase treatment (**B**,**b1**–**b3**). 1, 2, and 3 represent the mash of fermentation on the first, second, and third days, respectively. SEM photographs at 300× magnification are shown in (**A**,**B**); SEM photographs at 1000× magnification are shown in (**a1**–**a3**,**b1**–**b3**).

Figure 5 shows the scanning electron micrographs (SEM) of the pomace of Mopan persimmon wine at different magnifications (300× and 1000×). The results showed that the surface of pomace with pectinase was smooth with complete and uneven pores (Figure 5A), while the surface of pomace without pectinase was wrinkled and rough (Figure 5B). The SEM was magnified 1000× and many visible spherical particles were found on the surface of the fermentation residue, as shown in Figure 5(a1–a3, b1–b3). The EDS energy spectrum confirmed that the surface particles of the persimmon mash were mainly composed of C, O, and N elements. Combined with the fermentation process and particle shape, the granular substances were confirmed to be yeasts. According to the SEM images, the yeast particles in pomace with pectinase treatment increased gradually on the first, second, and third days of fermentation (Figure 5a), while the number of yeast particles in the pomace without pectinase treatment decreased gradually (Figure 5b). The pomace with pectinase treatment had a larger network structure, more pores, and more yeast than those without pectinase treatment. According to these SEM photographs and other results, it was confirmed that upon treatment with pectinase, the pectin in persimmon pulp was degraded, the viscosity of the pulp decreased, and the dissolved oxygen increased, promoting the proliferation of the yeast population, which was conducive to the conversion of sugar into alcohol.

### 3.5. Correlation Analysis

Table 3 shows the correlation analysis of chemical components in the fermentation process of Mopan persimmon wine with pectinase. There was a significant negative correlation between wine viscosity and alcohol content (*p* < 0.05) with a correlation coefficient of −0.854 and there was an extremely significant correlation between wine viscosity and pectin content (*p* < 0.01) with a correlation coefficient of 0.996. Pectinase decomposes pectin in persimmon mash, hydrolyzes pectin into soluble pectin, and disintegrates the cell wall and intercellular layer, by which the mash will seep out of the cell, reducing the viscosity of persimmon pulp and improving the quality of persimmon wine. There was a significant correlation between dissolved oxygen content and residual sugar content and total phenol content (*p* < 0.05) with correlation coefficients of −0.865 and 0.884, respectively. There was an extremely significant correlation between dissolved oxygen content and alcohol content (*p* < 0.01) with a correlation coefficient of 0.953. The increase in dissolved oxygen in the persimmon pulp promoted the fermentation process, which made more sugar convert into alcohol and increased the ethanol content of the persimmon wine. There was a significant negative correlation between the residual sugar and alcohol content and total phenol content (*p* < 0.01) with correlation coefficients of −0.915 and −0.921, respectively. In the fermentation process, yeast produces pyruvic acid by glycolysis and then decarboxylates pyruvic acid to acetaldehyde. Acetaldehyde is reduced to ethanol; thus, with the continuous decrease in sugar content, the degree of alcohol gradually increases.

## 4. Conclusions

In this study, a total of 427 single-colony yeast strains were screened from multiple persimmon samples; one strain was selected as the fermentation yeast for Mopan persimmon wine. The optimal conditions for fermentation of persimmon wine were determined to be yeast addition of 0.8 g/kg, fermentation temperature of 28 °C, sucrose addition of 18%, and pectinase addition of 0.10%. Under these conditions, the alcohol content can reach 12.90%. To determine the effect of pectinase on the fermentation process of the wine, the physicochemical properties and yeast growth status during the fermentation process were determined. The results showed that pectinase treatment significantly reduced the viscosity of the pulp at the initial stage of fermentation but did not affect the final viscosity of Mopan persimmon wine (5.7 mPa•S). According to the SEM photographs, the pomace with pectinase treatment had a larger network structure, more pores, and more yeast than those without pectinase treatment. By comparing the changes in physical and chemical indexes during the fermentation process of wine with and without pectinase, the addition of pectinase to the persimmon pulp could rapidly reduce the viscosity of the pulp, increase the dissolved oxygen content in the fermentation mash, and thus promote the growth of yeast. So, the sugar in the fermentation broth can be effectively converted into ethanol, increasing the alcohol content of Mopan persimmon wine and improving the fermentation efficiency.

## Figures and Tables

**Table 1:** Changes of soluble solids (°Brix) by screening 10 yeast strains.

Yeast Strain	0 d	1 d	2 d	3 d	4 d	5 d	6 d	Ethanol (%vol)
F1	15.42 ± 0.02 ^a^	12.85 ± 0.04 ^b^	10.73 ± 0.05 ^c^	9.34 ± 0.03 ^a^	8.18 ± 0.07 ^b^	7.06 ± 0.31 ^c^	6.94 ± 0.05 ^a^	5.25 ± 0.08 ^h^
F2	15.43 ± 0.03 ^a^	12.73 ± 0.13 ^d^	10.51 ± 0.08 ^d^	9.10 ± 0.04 ^d^	7.83 ± 0.03 ^e^	6.93 ± 0.01 ^e^	6.77 ± 0.40 ^e^	5.80 ± 0.09 ^c^
F3	15.40 ± 0.01 ^a^	11.98 ± 0.05 ^k^	9.68 ± 0.05 ^j^	8.99 ± 0.04 ^f^	7.69 ± 0.08 ^g^	6.58 ± 0.01 ^i^	6.46 ± 0.05 ^i^	5.56 ± 0.16 ^d^
F4	15.43 ± 0.03 ^a^	12.64 ± 0.21 ^f^	10.40 ± 0.03 ^e^	8.95 ± 0.01 ^g^	7.73 ± 0.18 ^f^	6.86 ± 0.03 ^g^	6.73 ± 0.05 ^f^	5.38 ± 0.02 ^f^
F5	15.42 ± 0.03 ^a^	12.88 ± 0.08 ^a^	10.84 ± 0.16 ^a^	9.24 ± 0.04 ^b^	8.20 ± 0.12 ^a^	7.09 ± 0.01 ^b^	6.85 ± 0.29 ^d^	5.25 ± 0.05 ^h^
F6	15.41 ± 0.00 a	12.00 ± 0.04 ^j^	9.74 ± 0.05 ^i^	8.07 ± 0.09 ^i^	7.54 ± 0.06 ^h^	6.90 ± 0.19 ^f^	6.90 ± 0.06 ^b^	5.31 ± 0.05 ^g^
F7	15.43 ± 0.03 ^a^	12.61 ± 0.09 ^g^	10.79 ± 0.08 ^b^	8.94 ± 0.14 ^h^	7.99 ± 0.10 ^d^	6.84 ± 0.12 ^h^	6.53 ± 0.07 ^h^	5.56 ± 0.11 ^d^
F8	15.44 ± 0.03 ^a^	12.75 ± 0.05 ^c^	10.40 ± 0.05 ^e^	9.22 ± 0.14 ^c^	8.03 ± 0.03 ^c^	7.18 ± 0.06 ^a^	6.86 ± 0.05 ^c^	5.31 ± 0.08 ^g^
F9	15.44 ± 0.02 ^a^	12.10 ± 0.14 ^h^	9.97 ± 0.05 ^g^	7.97 ± 0.09 ^j^	6.68 ± 0.02 ^i^	6.03 ± 0.03 ^j^	5.92 ± 0.12 ^j^	5.98 ± 0.09 ^b^
F10	15.43 ± 0.01 ^a^	12.67 ± 0.09 ^e^	10.33 ± 0.24 ^f^	9.02 ± 0.09 ^e^	8.03 ± 0.03 ^c^	6.98 ± 0.18 ^d^	6.70 ± 0.05 ^g^	5.44 ± 0.05 ^e^

Notes: Values are given as the means ± standard deviations (*n* = 3), and the different letters within the same column are significantly different (*p* < 0.05).

**Table 2:** Influence of temperature on the physical and chemical characteristics of the wine at the end of fermentation.

Temperature/°C	20	24	28	32	36
Soluble solids (°Brix)	12.17 ± 0.05 ^b^	9.03 ± 0.05 ^d^	8.23 ± 0.29 ^e^	11.46 ± 0.14 ^c^	14.47 ± 0.08 ^a^
Ethanol (%vol)	10.55 ± 0.02 ^c^	12.15 ± 0.03 ^b^	12.75 ± 0.02 ^a^	10.40 ± 0.04 ^d^	8.20 ± 0.04 ^e^
Reducing sugar (g/100 mL)	1.16 ± 0.36 ^b^	0.94 ± 0.20 ^c^	0.53 ± 0.53 ^d^	1.19 ± 0.07 ^b^	1.85 ± 0.20 ^a^
Total sugar (g/100 mL)	2.01 ± 0.10 ^d^	2.89 ± 0.58 ^b^	1.87 ± 0.39 ^e^	2.05 ± 0.20 ^c^	3.29 ± 0.26 ^a^
Titratable acid (g/L)	7.97 ± 0.28 ^d^	8.06 ± 0.09 ^c^	8.25 ± 0.19 ^b^	8.06 ± 0.19 ^c^	12.19 ± 0.09 ^a^

Values are given as the means ± standard deviations (*n* = 3), and the different letters within each row are significantly different (*p* < 0.05).

**Table 3:** Correlation analysis of chemical components during fermentation of Mopan persimmon wine with pectinase.

	Viscosity	Dissolved Oxygen	Yeast Growth	Residual Sugar	Ethanol	Pectin	Total Phenolic
Viscosity	1						
Dissolved oxygen	−0.691	1					
Yeast growth	0.190	0.150	1				
Residual sugar	0.724	−0.865 *	−0.073	1			
Ethanol	−0.854 *	0.953 **	0.109	−0.915 **	1		
Pectin	0.996 **	−0.683	0.136	0.698	−0.852 *	1	
Total phenolic	−0.473	0.884 *	0.345	−0.921 **	0.837 *	−0.456	1

Notes: * represents a significant correlation (*p* < 0.05); ** represents an extremely significant correlation (*p* < 0.01).

## Data Availability

The data are not publicly available due to privacy.
